# Reviewed article: Bastos ECL, Vidal NAC, Aragão LS, Costa GMAC, Silveira JAC. Marketing violations involving products that compete with breastfeeding in the vicinity of early childhood education centers and health centers: a cross-sectional study based on an audit of the retail food environment, Maceió, 2022-2023. Epidemiol Serv Saude. 2025:34;e20240666

**DOI:** 10.1590/S2237-96222025v34e20240666.c

**Published:** 2025-09-29

**Authors:** Carla Cristina Enes

**Affiliations:** 1 Pontifícia Universidade Católica de Campinas, Campinas, SP, Brazil Campinas SP Brazil

## First round comments

Prezados autores, o manuscrito foi bem avaliado pelos pareceristas, que destacaram a clareza da escrita, a objetividade da linguagem e a boa organização das ideias. No entanto, algumas revisões são necessárias para aprimorar a precisão e a consistência do trabalho. Dentre as sugestões estão incluir as definições das variáveis relacionadas às fórmulas infantis, garantindo que estejam alinhadas com a legislação vigente e com as denominações das marcas presentes nas embalagens. Além disso, foi apontada a necessidade de incluir o nome do fabricante como uma variável de interesse no tópico Métodos. Foi recomendada a inclusão de uma referência específica para apoiar o resultado sobre a maior frequência de infrações em estabelecimentos pertencentes à rede. Sugere-se ainda que o título seja mais objetivo. Exemplificar os avanços e índices mencionados na Introdução e citar o impacto na evolução dos indicadores. Recomenda-se ainda ajustes na Figura 1 e na Tabela 1.

Recommendation: Minor revision

